# Supplementary material for: Modeling glioblastoma heterogeneity as a dynamic network of cell states
Source: Mol Syst Biol. 2021 Sep 16;17(9):e10105. doi: 10.15252/msb.202010105 (PMC8444284; doi:10.15252/msb.202010105)
Supplement: Supplementary file 6 — Source Data for Figure 5 [file MSB-17-e10105-s004.zip › Figure5A_sourcedata/GSEA_3017/nosigdb_stateF.GseaPreranked.1622795015644/RICHARDS_NOWAKOWSKI_SCIENCE_2017_ASTROCYTE_UPREG.html]

Details for gene set RICHARDS\_NOWAKOWSKI\_SCIENCE\_2017\_ASTROCYTE\_UPREG[GSEA]

|  || Dataset | state33017 |
| Phenotype | NoPhenotypeAvailable |
| Upregulated in class | na\_pos |
| GeneSet | RICHARDS\_NOWAKOWSKI\_SCIENCE\_2017\_ASTROCYTE\_UPREG |
| Enrichment Score (ES) | 0.465346 |
| Normalized Enrichment Score (NES) | 3.4732857 |
| Nominal p-value | 0.0 |
| FDR q-value | 0.0 |
| FWER p-Value | 0.0 |
Table: GSEA Results Summary

  

Fig 1: Enrichment plot: RICHARDS\_NOWAKOWSKI\_SCIENCE\_2017\_ASTROCYTE\_UPREG      
 Profile of the Running ES Score & Positions of GeneSet Members on the Rank Ordered List

  

| PROBE | GENE SYMBOL | GENE\_TITLE | RANK IN GENE LIST | RANK METRIC SCORE | RUNNING ES | CORE ENRICHMENT || 1 | NEAT1 |  |  | 0 | 2.864 | 0.0214 | Yes |
| 2 | MT3 |  |  | 1 | 2.229 | 0.0380 | Yes |
| 3 | CLU |  |  | 5 | 1.852 | 0.0504 | Yes |
| 4 | SPARCL1 |  |  | 6 | 1.804 | 0.0638 | Yes |
| 5 | ATP1B2 |  |  | 9 | 1.759 | 0.0760 | Yes |
| 6 | ZMAT3 |  |  | 10 | 1.568 | 0.0877 | Yes |
| 7 | HEPACAM |  |  | 13 | 1.356 | 0.0968 | Yes |
| 8 | HOPX |  |  | 14 | 1.345 | 0.1068 | Yes |
| 9 | CST3 |  |  | 15 | 1.273 | 0.1163 | Yes |
| 10 | S100B |  |  | 20 | 1.167 | 0.1231 | Yes |
| 11 | SLC1A3 |  |  | 26 | 1.058 | 0.1284 | Yes |
| 12 | CPE |  |  | 29 | 1.046 | 0.1352 | Yes |
| 13 | CSPG5 |  |  | 30 | 1.044 | 0.1430 | Yes |
| 14 | LRRC17 |  |  | 31 | 1.044 | 0.1508 | Yes |
| 15 | ITPR2 |  |  | 34 | 1.024 | 0.1575 | Yes |
| 16 | SLC4A4 |  |  | 40 | 0.980 | 0.1623 | Yes |
| 17 | F3 |  |  | 41 | 0.978 | 0.1696 | Yes |
| 18 | EDNRB |  |  | 44 | 0.957 | 0.1757 | Yes |
| 19 | SAT1 |  |  | 45 | 0.957 | 0.1829 | Yes |
| 20 | GFAP |  |  | 47 | 0.953 | 0.1895 | Yes |
| 21 | CXCL14 |  |  | 51 | 0.931 | 0.1949 | Yes |
| 22 | SESN3 |  |  | 56 | 0.908 | 0.1997 | Yes |
| 23 | MLC1 |  |  | 58 | 0.896 | 0.2059 | Yes |
| 24 | MYO6 |  |  | 62 | 0.859 | 0.2108 | Yes |
| 25 | SLC1A2 |  |  | 63 | 0.857 | 0.2172 | Yes |
| 26 | GLUL |  |  | 69 | 0.837 | 0.2209 | Yes |
| 27 | AGT |  |  | 72 | 0.820 | 0.2260 | Yes |
| 28 | CHPT1 |  |  | 73 | 0.818 | 0.2322 | Yes |
| 29 | MPC1 |  |  | 78 | 0.804 | 0.2361 | Yes |
| 30 | AQP4 |  |  | 84 | 0.789 | 0.2395 | Yes |
| 31 | UBL3 |  |  | 88 | 0.778 | 0.2438 | Yes |
| 32 | MALAT1 |  |  | 90 | 0.773 | 0.2491 | Yes |
| 33 | LRP1 |  |  | 92 | 0.765 | 0.2543 | Yes |
| 34 | PON2 |  |  | 93 | 0.764 | 0.2600 | Yes |
| 35 | GRIA4 |  |  | 99 | 0.754 | 0.2631 | Yes |
| 36 | RNF180 |  |  | 101 | 0.751 | 0.2682 | Yes |
| 37 | NDRG4 |  |  | 104 | 0.747 | 0.2728 | Yes |
| 38 | LUZP2 |  |  | 106 | 0.742 | 0.2778 | Yes |
| 39 | LTBP1 |  |  | 108 | 0.741 | 0.2829 | Yes |
| 40 | B2M |  |  | 110 | 0.736 | 0.2879 | Yes |
| 41 | GNG7 |  |  | 115 | 0.726 | 0.2913 | Yes |
| 42 | NPNT |  |  | 117 | 0.721 | 0.2962 | Yes |
| 43 | CADM2 |  |  | 137 | 0.687 | 0.2917 | Yes |
| 44 | NCAN |  |  | 138 | 0.686 | 0.2969 | Yes |
| 45 | TSPAN7 |  |  | 148 | 0.671 | 0.2973 | Yes |
| 46 | DDR1 |  |  | 159 | 0.649 | 0.2971 | Yes |
| 47 | ITM2C |  |  | 160 | 0.649 | 0.3020 | Yes |
| 48 | APC |  |  | 169 | 0.632 | 0.3027 | Yes |
| 49 | CSGALNACT1 |  |  | 172 | 0.628 | 0.3064 | Yes |
| 50 | DTNA |  |  | 176 | 0.617 | 0.3095 | Yes |
| 51 | ST3GAL5 |  |  | 180 | 0.612 | 0.3125 | Yes |
| 52 | ITM2B |  |  | 181 | 0.610 | 0.3171 | Yes |
| 53 | PLOD2 |  |  | 183 | 0.607 | 0.3211 | Yes |
| 54 | MTRNR2L8 |  |  | 186 | 0.605 | 0.3246 | Yes |
| 55 | KAT2B |  |  | 193 | 0.594 | 0.3260 | Yes |
| 56 | TIMP2 |  |  | 196 | 0.590 | 0.3294 | Yes |
| 57 | C1orf61 |  |  | 202 | 0.587 | 0.3313 | Yes |
| 58 | ENHO |  |  | 203 | 0.586 | 0.3357 | Yes |
| 59 | GPR37L1 |  |  | 220 | 0.576 | 0.3319 | Yes |
| 60 | PDLIM5 |  |  | 223 | 0.572 | 0.3352 | Yes |
| 61 | KCNJ16 |  |  | 227 | 0.569 | 0.3379 | Yes |
| 62 | HTRA1 |  |  | 229 | 0.568 | 0.3417 | Yes |
| 63 | LIFR |  |  | 239 | 0.562 | 0.3414 | Yes |
| 64 | MEGF10 |  |  | 240 | 0.560 | 0.3455 | Yes |
| 65 | CA2 |  |  | 243 | 0.558 | 0.3487 | Yes |
| 66 | PHYHIPL |  |  | 245 | 0.557 | 0.3524 | Yes |
| 67 | PBXIP1 |  |  | 250 | 0.554 | 0.3545 | Yes |
| 68 | S100A13 |  |  | 261 | 0.544 | 0.3535 | Yes |
| 69 | FAM3C |  |  | 266 | 0.541 | 0.3555 | Yes |
| 70 | PRNP |  |  | 269 | 0.538 | 0.3585 | Yes |
| 71 | HMGCS1 |  |  | 271 | 0.537 | 0.3620 | Yes |
| 72 | LRP1B |  |  | 276 | 0.530 | 0.3640 | Yes |
| 73 | FAM19A5 |  |  | 277 | 0.527 | 0.3679 | Yes |
| 74 | PCDH9 |  |  | 278 | 0.527 | 0.3719 | Yes |
| 75 | SLC25A18 |  |  | 280 | 0.525 | 0.3753 | Yes |
| 76 | BCAN |  |  | 302 | 0.510 | 0.3685 | Yes |
| 77 | SCD5 |  |  | 306 | 0.508 | 0.3708 | Yes |
| 78 | PTPRZ1 |  |  | 310 | 0.505 | 0.3731 | Yes |
| 79 | HES1 |  |  | 320 | 0.501 | 0.3723 | Yes |
| 80 | IL6ST |  |  | 321 | 0.500 | 0.3760 | Yes |
| 81 | PSAT1 |  |  | 329 | 0.496 | 0.3762 | Yes |
| 82 | RNF13 |  |  | 330 | 0.496 | 0.3799 | Yes |
| 83 | GPC6 |  |  | 332 | 0.495 | 0.3831 | Yes |
| 84 | SEMA5A |  |  | 336 | 0.494 | 0.3853 | Yes |
| 85 | DCLK2 |  |  | 340 | 0.492 | 0.3874 | Yes |
| 86 | OLFM2 |  |  | 343 | 0.490 | 0.3901 | Yes |
| 87 | ITGAV |  |  | 344 | 0.490 | 0.3937 | Yes |
| 88 | ACSBG1 |  |  | 345 | 0.490 | 0.3974 | Yes |
| 89 | SYT11 |  |  | 352 | 0.486 | 0.3980 | Yes |
| 90 | SCP2 |  |  | 353 | 0.485 | 0.4016 | Yes |
| 91 | ABAT |  |  | 362 | 0.483 | 0.4012 | Yes |
| 92 | ID2 |  |  | 366 | 0.483 | 0.4033 | Yes |
| 93 | CADM4 |  |  | 368 | 0.482 | 0.4064 | Yes |
| 94 | GATM |  |  | 375 | 0.480 | 0.4070 | Yes |
| 95 | SEMA6A |  |  | 381 | 0.478 | 0.4080 | Yes |
| 96 | LRRTM3 |  |  | 387 | 0.476 | 0.4091 | Yes |
| 97 | MTSS1L |  |  | 389 | 0.475 | 0.4121 | Yes |
| 98 | TGFB2 |  |  | 390 | 0.474 | 0.4156 | Yes |
| 99 | COMT |  |  | 396 | 0.471 | 0.4166 | Yes |
| 100 | METTL7A |  |  | 403 | 0.469 | 0.4171 | Yes |
| 101 | ADI1 |  |  | 415 | 0.464 | 0.4151 | Yes |
| 102 | PSAP |  |  | 418 | 0.463 | 0.4175 | Yes |
| 103 | DNASE2 |  |  | 420 | 0.462 | 0.4205 | Yes |
| 104 | DPP6 |  |  | 421 | 0.462 | 0.4239 | Yes |
| 105 | PRCP |  |  | 426 | 0.460 | 0.4253 | Yes |
| 106 | NADK2 |  |  | 437 | 0.456 | 0.4237 | Yes |
| 107 | TRPS1 |  |  | 441 | 0.455 | 0.4256 | Yes |
| 108 | MAST4 |  |  | 446 | 0.451 | 0.4269 | Yes |
| 109 | HSD17B12 |  |  | 453 | 0.449 | 0.4273 | Yes |
| 110 | TTYH1 |  |  | 457 | 0.448 | 0.4291 | Yes |
| 111 | METTL8 |  |  | 458 | 0.448 | 0.4325 | Yes |
| 112 | EFHD2 |  |  | 464 | 0.445 | 0.4333 | Yes |
| 113 | FHL1 |  |  | 467 | 0.445 | 0.4356 | Yes |
| 114 | ZNF521 |  |  | 476 | 0.442 | 0.4349 | Yes |
| 115 | RFTN2 |  |  | 485 | 0.439 | 0.4341 | Yes |
| 116 | LPIN2 |  |  | 488 | 0.438 | 0.4364 | Yes |
| 117 | HEATR5A |  |  | 491 | 0.438 | 0.4387 | Yes |
| 118 | GJA1 |  |  | 495 | 0.436 | 0.4404 | Yes |
| 119 | HEY1 |  |  | 496 | 0.436 | 0.4437 | Yes |
| 120 | NDUFB5 |  |  | 498 | 0.435 | 0.4464 | Yes |
| 121 | AHCYL2 |  |  | 508 | 0.432 | 0.4451 | Yes |
| 122 | FGFR3 |  |  | 515 | 0.429 | 0.4453 | Yes |
| 123 | PEA15 |  |  | 521 | 0.427 | 0.4460 | Yes |
| 124 | LAMB2 |  |  | 524 | 0.425 | 0.4481 | Yes |
| 125 | ELOVL2 |  |  | 527 | 0.424 | 0.4503 | Yes |
| 126 | LGALS3BP |  |  | 528 | 0.424 | 0.4535 | Yes |
| 127 | TFPI |  |  | 531 | 0.421 | 0.4556 | Yes |
| 128 | TMEM9B |  |  | 539 | 0.417 | 0.4552 | Yes |
| 129 | PARP14 |  |  | 541 | 0.417 | 0.4578 | Yes |
| 130 | RASSF2 |  |  | 544 | 0.417 | 0.4599 | Yes |
| 131 | GPM6B |  |  | 553 | 0.413 | 0.4590 | Yes |
| 132 | PMP2 |  |  | 562 | 0.411 | 0.4580 | Yes |
| 133 | IGFBP5 |  |  | 564 | 0.410 | 0.4606 | Yes |
| 134 | CNN3 |  |  | 576 | 0.407 | 0.4581 | Yes |
| 135 | RAB31 |  |  | 580 | 0.404 | 0.4596 | Yes |
| 136 | SLC6A1 |  |  | 582 | 0.404 | 0.4621 | Yes |
| 137 | FOS |  |  | 592 | 0.401 | 0.4605 | Yes |
| 138 | ARAP2 |  |  | 599 | 0.399 | 0.4605 | Yes |
| 139 | LSAMP |  |  | 611 | 0.394 | 0.4579 | Yes |
| 140 | TNC |  |  | 613 | 0.393 | 0.4603 | Yes |
| 141 | SASH1 |  |  | 614 | 0.393 | 0.4633 | Yes |
| 142 | NTRK3 |  |  | 621 | 0.391 | 0.4632 | Yes |
| 143 | NDUFB3 |  |  | 629 | 0.389 | 0.4626 | Yes |
| 144 | TAOK3 |  |  | 631 | 0.389 | 0.4650 | Yes |
| 145 | SEMA6D |  |  | 637 | 0.388 | 0.4653 | Yes |
| 146 | GPRC5B |  |  | 653 | 0.385 | 0.4607 | No |
| 147 | SEZ6L |  |  | 657 | 0.383 | 0.4620 | No |
| 148 | ID4 |  |  | 659 | 0.383 | 0.4644 | No |
| 149 | ADD3 |  |  | 674 | 0.378 | 0.4601 | No |
| 150 | BCHE |  |  | 677 | 0.376 | 0.4619 | No |
| 151 | CCDC80 |  |  | 683 | 0.375 | 0.4622 | No |
| 152 | NDUFA4 |  |  | 698 | 0.371 | 0.4580 | No |
| 153 | FABP7 |  |  | 701 | 0.371 | 0.4597 | No |
| 154 | FAM107A |  |  | 718 | 0.367 | 0.4544 | No |
| 155 | ZDHHC21 |  |  | 728 | 0.364 | 0.4526 | No |
| 156 | SC5D |  |  | 737 | 0.362 | 0.4513 | No |
| 157 | ALDH1L1 |  |  | 744 | 0.361 | 0.4510 | No |
| 158 | MASP1 |  |  | 755 | 0.357 | 0.4486 | No |
| 159 | FGF1 |  |  | 771 | 0.353 | 0.4437 | No |
| 160 | LRP4 |  |  | 773 | 0.353 | 0.4458 | No |
| 161 | PTPRA |  |  | 777 | 0.352 | 0.4469 | No |
| 162 | RORA |  |  | 783 | 0.351 | 0.4470 | No |
| 163 | SLC15A2 |  |  | 784 | 0.351 | 0.4497 | No |
| 164 | C3orf70 |  |  | 794 | 0.348 | 0.4477 | No |
| 165 | FAM84B |  |  | 804 | 0.346 | 0.4458 | No |
| 166 | BDH2 |  |  | 806 | 0.346 | 0.4479 | No |
| 167 | PPARGC1A |  |  | 821 | 0.343 | 0.4434 | No |
| 168 | PLEKHH2 |  |  | 822 | 0.343 | 0.4459 | No |
| 169 | DAG1 |  |  | 834 | 0.340 | 0.4429 | No |
| 170 | VEGFA |  |  | 840 | 0.339 | 0.4430 | No |
| 171 | PCDHGC3 |  |  | 845 | 0.337 | 0.4435 | No |
| 172 | RCN1 |  |  | 849 | 0.337 | 0.4445 | No |
| 173 | ZFP36L2 |  |  | 850 | 0.336 | 0.4470 | No |
| 174 | CLSTN1 |  |  | 857 | 0.334 | 0.4465 | No |
| 175 | MSMO1 |  |  | 860 | 0.333 | 0.4479 | No |
| 176 | SDC3 |  |  | 863 | 0.333 | 0.4494 | No |
| 177 | CSMD1 |  |  | 870 | 0.331 | 0.4489 | No |
| 178 | RHOJ |  |  | 874 | 0.331 | 0.4498 | No |
| 179 | STAT3 |  |  | 880 | 0.330 | 0.4498 | No |
| 180 | WSB1 |  |  | 882 | 0.329 | 0.4517 | No |
| 181 | ARHGEF26 |  |  | 885 | 0.328 | 0.4532 | No |
| 182 | CRYL1 |  |  | 891 | 0.327 | 0.4531 | No |
| 183 | DST |  |  | 896 | 0.327 | 0.4535 | No |
| 184 | GSTM4 |  |  | 909 | 0.324 | 0.4499 | No |
| 185 | IGDCC4 |  |  | 911 | 0.324 | 0.4518 | No |
| 186 | ACOX1 |  |  | 912 | 0.324 | 0.4542 | No |
| 187 | ADCYAP1R1 |  |  | 913 | 0.324 | 0.4567 | No |
| 188 | IFI6 |  |  | 922 | 0.323 | 0.4550 | No |
| 189 | NKAIN4 |  |  | 923 | 0.322 | 0.4575 | No |
| 190 | P2RY1 |  |  | 939 | 0.319 | 0.4523 | No |
| 191 | CD81 |  |  | 947 | 0.318 | 0.4511 | No |
| 192 | ARHGAP31 |  |  | 951 | 0.317 | 0.4520 | No |
| 193 | SLITRK2 |  |  | 967 | 0.315 | 0.4468 | No |
| 194 | FAT1 |  |  | 968 | 0.315 | 0.4492 | No |
| 195 | ITGB8 |  |  | 970 | 0.314 | 0.4510 | No |
| 196 | ZMAT1 |  |  | 983 | 0.313 | 0.4473 | No |
| 197 | ALDH6A1 |  |  | 987 | 0.311 | 0.4481 | No |
| 198 | FMNL2 |  |  | 1005 | 0.309 | 0.4419 | No |
| 199 | SRGAP2B |  |  | 1006 | 0.309 | 0.4442 | No |
| 200 | RGMA |  |  | 1010 | 0.308 | 0.4450 | No |
| 201 | SERPINE2 |  |  | 1030 | 0.304 | 0.4377 | No |
| 202 | SCRG1 |  |  | 1032 | 0.304 | 0.4394 | No |
| 203 | CTNND2 |  |  | 1048 | 0.300 | 0.4341 | No |
| 204 | ARHGEF6 |  |  | 1054 | 0.299 | 0.4338 | No |
| 205 | NRBP2 |  |  | 1060 | 0.298 | 0.4336 | No |
| 206 | CD9 |  |  | 1070 | 0.297 | 0.4312 | No |
| 207 | TSPAN3 |  |  | 1071 | 0.297 | 0.4335 | No |
| 208 | SPECC1 |  |  | 1089 | 0.294 | 0.4271 | No |
| 209 | LYPD1 |  |  | 1094 | 0.293 | 0.4273 | No |
| 210 | DNER |  |  | 1104 | 0.292 | 0.4249 | No |
| 211 | PIK3R1 |  |  | 1121 | 0.289 | 0.4190 | No |
| 212 | SLC7A11 |  |  | 1131 | 0.288 | 0.4167 | No |
| 213 | SYPL1 |  |  | 1137 | 0.287 | 0.4163 | No |
| 214 | SNX3 |  |  | 1150 | 0.285 | 0.4124 | No |
| 215 | CACNB2 |  |  | 1155 | 0.285 | 0.4125 | No |
| 216 | SEPT8 |  |  | 1167 | 0.283 | 0.4091 | No |
| 217 | ZBTB20 |  |  | 1181 | 0.281 | 0.4046 | No |
| 218 | SLC35A5 |  |  | 1184 | 0.281 | 0.4057 | No |
| 219 | CTNNB1 |  |  | 1190 | 0.280 | 0.4053 | No |
| 220 | RGS20 |  |  | 1197 | 0.279 | 0.4043 | No |
| 221 | JAM2 |  |  | 1204 | 0.279 | 0.4034 | No |
| 222 | MAPK1 |  |  | 1250 | 0.274 | 0.3828 | No |
| 223 | CHP1 |  |  | 1251 | 0.274 | 0.3848 | No |
| 224 | ALDH7A1 |  |  | 1255 | 0.273 | 0.3854 | No |
| 225 | NBPF14 |  |  | 1258 | 0.273 | 0.3864 | No |
| 226 | SALL2 |  |  | 1274 | 0.271 | 0.3809 | No |
| 227 | SLC6A9 |  |  | 1278 | 0.271 | 0.3814 | No |
| 228 | NCKAP5 |  |  | 1279 | 0.271 | 0.3834 | No |
| 229 | NLGN3 |  |  | 1292 | 0.269 | 0.3794 | No |
| 230 | SLC18B1 |  |  | 1303 | 0.268 | 0.3764 | No |
| 231 | ABHD4 |  |  | 1313 | 0.267 | 0.3738 | No |
| 232 | RFX4 |  |  | 1320 | 0.266 | 0.3728 | No |
| 233 | GLUD1 |  |  | 1334 | 0.264 | 0.3682 | No |
| 234 | TTC14 |  |  | 1336 | 0.263 | 0.3697 | No |
| 235 | PARP9 |  |  | 1344 | 0.262 | 0.3681 | No |
| 236 | TRIM47 |  |  | 1353 | 0.262 | 0.3660 | No |
| 237 | SLCO1C1 |  |  | 1372 | 0.260 | 0.3589 | No |
| 238 | NRXN1 |  |  | 1374 | 0.259 | 0.3604 | No |
| 239 | ABCD3 |  |  | 1381 | 0.259 | 0.3593 | No |
| 240 | EEPD1 |  |  | 1397 | 0.257 | 0.3536 | No |
| 241 | WSCD1 |  |  | 1401 | 0.256 | 0.3540 | No |
| 242 | PDE4B |  |  | 1404 | 0.256 | 0.3549 | No |
| 243 | OPHN1 |  |  | 1405 | 0.256 | 0.3569 | No |
| 244 | MEIS1 |  |  | 1407 | 0.256 | 0.3583 | No |
| 245 | NOTCH2 |  |  | 1420 | 0.255 | 0.3541 | No |
| 246 | GLDC |  |  | 1425 | 0.255 | 0.3540 | No |
| 247 | PTAR1 |  |  | 1441 | 0.254 | 0.3484 | No |
| 248 | KANK1 |  |  | 1449 | 0.252 | 0.3467 | No |
| 249 | PEX2 |  |  | 1463 | 0.251 | 0.3420 | No |
| 250 | BBS2 |  |  | 1472 | 0.250 | 0.3399 | No |
| 251 | HNRNPA2B1 |  |  | 1503 | -0.261 | 0.3267 | No |
| 252 | PURB |  |  | 1527 | -0.267 | 0.3172 | No |
| 253 | ZFP36L1 |  |  | 1590 | -0.284 | 0.2881 | No |
| 254 | LHX2 |  |  | 1597 | -0.285 | 0.2872 | No |
| 255 | PDGFRA |  |  | 1601 | -0.286 | 0.2878 | No |
| 256 | ILDR2 |  |  | 1650 | -0.300 | 0.2659 | No |
| 257 | ENO1 |  |  | 1673 | -0.308 | 0.2571 | No |
| 258 | TACC1 |  |  | 1701 | -0.319 | 0.2459 | No |
| 259 | UGP2 |  |  | 1725 | -0.327 | 0.2368 | No |
| 260 | DSEL |  |  | 1735 | -0.333 | 0.2347 | No |
| 261 | RAB13 |  |  | 1761 | -0.345 | 0.2247 | No |
| 262 | LGALS1 |  |  | 1799 | -0.362 | 0.2088 | No |
| 263 | CREM |  |  | 1842 | -0.385 | 0.1905 | No |
| 264 | PTCH1 |  |  | 1857 | -0.393 | 0.1864 | No |
| 265 | LIMA1 |  |  | 1899 | -0.421 | 0.1689 | No |
| 266 | FABP5 |  |  | 2033 | -0.523 | 0.1059 | No |
| 267 | EPB41L2 |  |  | 2067 | -0.576 | 0.0936 | No |
Table: GSEA details [plain text format]

  

Fig 2: RICHARDS\_NOWAKOWSKI\_SCIENCE\_2017\_ASTROCYTE\_UPREG: Random ES distribution      
 Gene set null distribution of ES for **RICHARDS\_NOWAKOWSKI\_SCIENCE\_2017\_ASTROCYTE\_UPREG**
